# Development and Validation of a Radiomics Model Based on ^18^F-FDG PET of Primary Gastric Cancer for Predicting Peritoneal Metastasis

**DOI:** 10.3389/fonc.2021.740111

**Published:** 2021-10-26

**Authors:** Beihui Xue, Jia Jiang, Lei Chen, Sunjie Wu, Xuan Zheng, Xiangwu Zheng, Kun Tang

**Affiliations:** ^1^ Department of Radiology, The First Affiliated Hospital of Wenzhou Medical University, Wenzhou, China; ^2^ Department of Nuclear Medicine, The First Affiliated Hospital of Wenzhou Medical University, Wenzhou, China

**Keywords:** gastric cancer, peritoneal metastasis, positron emission tomography radiomics (PET radiomics), positron emission tomography/computed tomography (PET/CT), nomogram

## Abstract

**Objectives:**

The aim of this study was to develop a preoperative positron emission tomography (PET)-based radiomics model for predicting peritoneal metastasis (PM) of gastric cancer (GC).

**Methods:**

In this study, a total of 355 patients (109PM+, 246PM-) who underwent preoperative fluorine-18-fludeoxyglucose (^18^F-FDG) PET images were retrospectively analyzed. According to a 7:3 ratio, patients were randomly divided into a training set and a validation set. Radiomics features and metabolic parameters data were extracted from PET images. The radiomics features were selected by logistic regression after using maximum relevance and minimum redundancy (mRMR) and the least shrinkage and selection operator (LASSO) method. The radiomics models were based on the rest of these features. The performance of the models was determined by their discrimination, calibration, and clinical usefulness in the training and validation sets.

**Results:**

After dimensionality reduction, 12 radiomics feature parameters were obtained to construct radiomics signatures. According to the results of the multivariate logistic regression analysis, only carbohydrate antigen 125 (CA125), maximum standardized uptake value (SUVmax), and the radiomics signature showed statistically significant differences between patients (P<0.05). A radiomics model was developed based on the logistic analyses with an AUC of 0.86 in the training cohort and 0.87 in the validation cohort. The clinical prediction model based on CA125 and SUVmax was 0.76 in the training set and 0.69 in the validation set. The comprehensive model, which contained a rad-score and the clinical factor (CA125) as well as the metabolic parameter (SUVmax), showed promising performance with an AUC of 0.90 in the training cohort and 0.88 in the validation cohort, respectively. The calibration curve showed the actual rate of the nomogram-predicted probability of peritoneal metastasis. Decision curve analysis (DCA) also demonstrated the good clinical utility of the radiomics nomogram.

**Conclusions:**

The comprehensive model based on the rad-score and other factors (SUVmax, CA125) can provide a novel tool for predicting peritoneal metastasis of gastric cancer patients preoperatively.

## Introduction

Gastric cancer (GC) is the fifth most frequent type of cancer and the third-leading cause of cancer-related death worldwide (1). Over the last decades, its incidence and mortality have decreased. However, East Asia including China still has the highest mortality rate (2). Generally, because early-stage GC is commonly asymptomatic, this causes most GC patients to be initially diagnosed at the advanced stage (3). Therefore, the prognosis of patients with GC remains poor, and the 5-year overall survival rate is only 40–60% in Asia and 24.5% in Europe (4, 5).

Among the GC patients, the most frequent form of metastasis is peritoneal metastasis (PM). Moreover, PM is the primary factor leading to the decrease in survival time in patients with GC (6). The presence of PM had a profound negative impact on survival with a median survival of only 4 months (7). Therefore, accurate assessment of the PM status of GC patients is important for treatment and prognosis. Computed tomography (CT) is a common method in the diagnosis of GC, but its sensitivity in the evaluation of PM is low (8). ^18^F-fluorodeoxyglucose positron emission tomography/CT (^18^F-FDG PET/CT) is a powerful, noninvasive tool to evaluate various tumors (9–11). The sensitivity of detecting PM by PET/CT is higher than CT (12). However, most imaging information is not visible to the naked eye. Instead, radiomics is an approach that can provide complementary data on imaging.

Radiomics, a newly developed field that involves a great quantity of data, has attracted increasing attention in recent years (13, 14). The emerging field of “radiomics” has great potential in disease diagnosis, prognosis evaluation, and prediction of treatment (15). It successfully showed favorable abilities in clinical management (16–19). However, no studies have used the PET-based radiomics tool to predicting the PM of GC.

In this study, we attempt to further explore a novel model based on ^18^F-FDG PET combined with clinical and metabolic factors to predict the PM of GC.

## Methods

### Patient Selection

**Figure 1:**
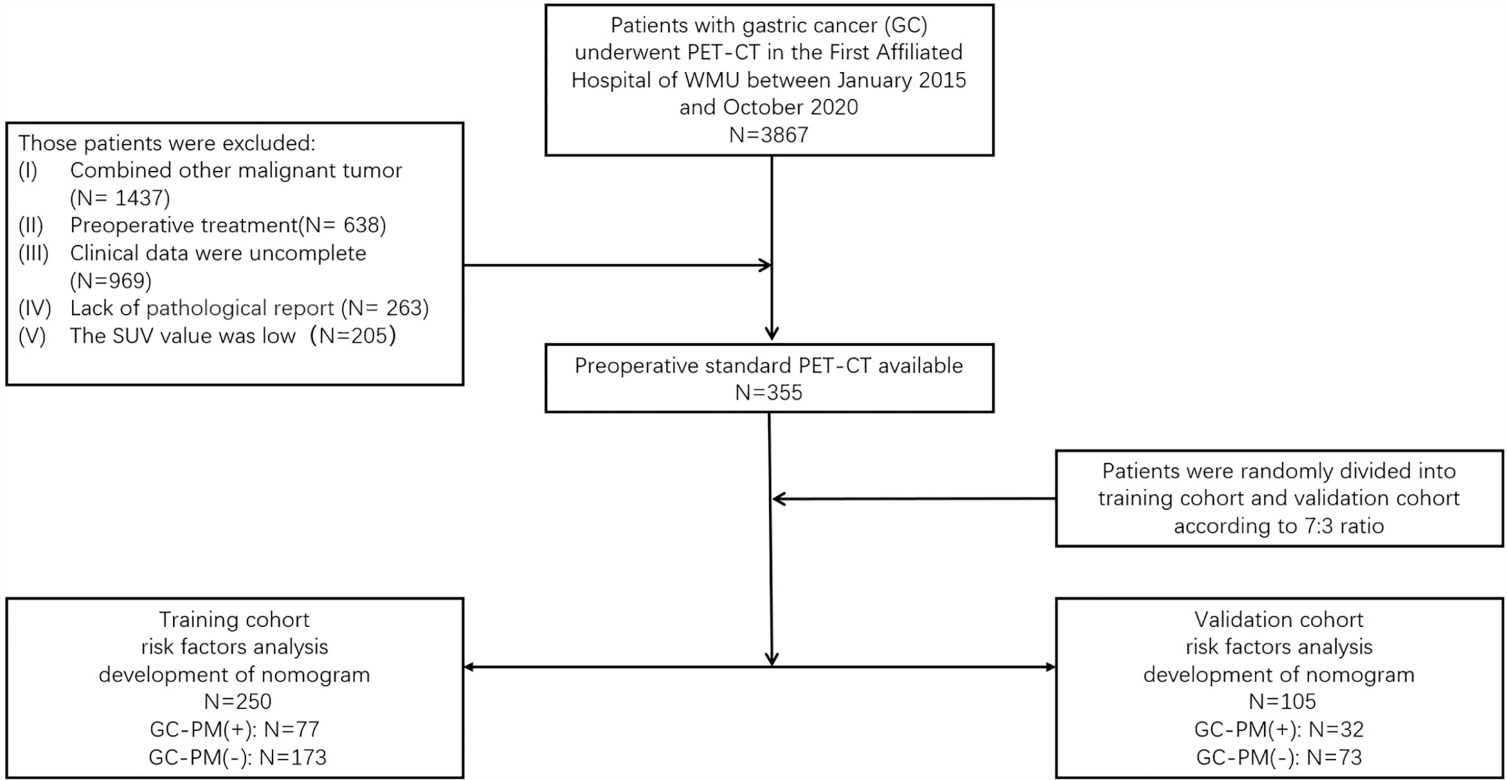
Proceeding flow of enrollment.

The ethics committee of the First Affiliated Hospital of Wenzhou Medical University (WMU) approved this retrospective analysis and waived the requirement to obtain informed consent from the patients (2021R061). All patients were involved in this study from January 2015 to October 2020. The potentially eligible patients were as follows: (I) underwent PET/CT examination and (II) confirmed by operation and the pathology proved they have PM. The criteria for excluding patients were as follows: (I) combined with other malignant tumor, (II) preoperative treatment, (III) clinical data were incomplete, (IV) lack of pathological report, and (V) the standardized uptake value (SUV) was low. A total of 355 patients with gastric cancer confirmed by endoscopy and pathology were included. The histological and pathological classifications of gastric cancer in this study were all gastric carcinoma, including tubular adenocarcinoma, mucinous adenocarcinoma, papillary adenocarcinoma, and signet-ring cell carcinoma. Among all the patients, 166 had a surgery. The type of surgery included total gastrectomy (20), endoscopic mucosal resection (21), distal esophagectomy (18), and subtotal gastrectomy (96). Moreover, 109 patients had their tissues biopsied, which were confirmed by the pathology that proved they have PM. Patients were randomly divided into a training set and a validation set according to the 7:3 ratio (21). Their clinical data, including age, gender, smoking, alcohol, gastric ulcers, symptoms (abdominal pain, fever, vomit, weight loss), and the serum carcinoembryonic antigen (CEA) level and CA125 and carbohydrate antigen 199 (CA199) level were marked (**Figure 1**).

### PET/CT Image Acquisition

After at least 6 h of fasting, the patients received an intravenous injection of ^18^F-FDG (3.7 MBq/kg). Blood glucose was controlled below 110 ml/dl. Approximately 60 min later, images were acquired by a hybrid PET/CT scanner (GEMINI TF 64, Philips, Netherlands). Subsequently, a 3D model was used to obtain PET images. The parameters were set as follows: field of view of 576 mm, a matrix of 144×144, slice thickness and interval of 5 mm, and an emission scan time of each bed position of 1.5 min. PET images with CT attenuation correction were reconstructed using the time-of-flight algorithm.

### Tumor Segmentation

**Figure 2:**
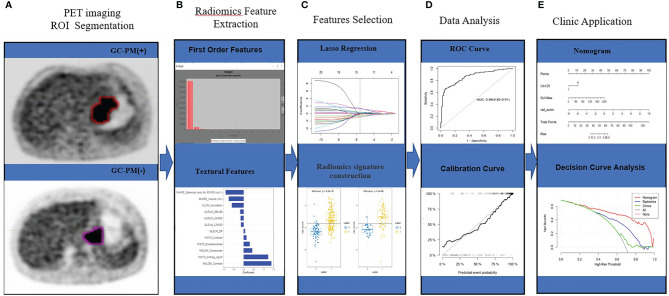
Workflow. **(A)** PET imaging ROI segmentation. **(B)** Radiomic features are extracted by the LIFEx software with qualified tumor intensity, shape, and texture. **(C)** PET feature selection using the mRMR and LASSO regularization. Rad-score was using Wilcoxon analysis for detecting the PM of GC. **(D)** The performance of the prediction model is assessed by the area under a receiver operating characteristic (ROC) curve and the calibration curve. **(E)** The PET radiomics nomogram with SUVmax, CA125, and radiomics signatures. Decision curve analysis for radiomics signatures.

The radiomics workflow is depicted in **Figure 2**. The region of interest (ROI) segmentation of tumors was semiautomatically produced by LIFEX software tools (22) by two radiologists with a great wealth of clinical diagnosis experience. ^18^FDG-PET images were read by software using the digital imaging and communications in medicine (DICOM) protocol. The SUVmax of gastric target lesions was automatically measured, and the ROI was delineated by the LIFEX software program. Similarly, the metabolic tumor volume (MTV) and total lesion glycolysis (TLG) of the gastric target lesions were also automatically measured, and the volume of interest was delineated with a threshold of 40% of SUVmax.

### Radiomics Feature Extraction, Selection, and Signature Construction

We extracted a total of 69 quantified texture features from the LIFEX software. The first order involved measuring a shape-based matrix and a histogram‐based matrix. The second or higher order included a gray-level co-occurrence matrix (GLCM), a gray-level zone length matrix (GLZLM), a neighborhood gray-level dependence matrix (NGLDM), and a gray-level run length matrix (GLRLM) (23).

With the abundance of radiomics features, feature selection was essential to deliver the most optimal predictive features. In order to reduce the dimensionality, we devised a two-step procedure. First, interclass and intraclass correlation coefficients (ICCs) were calculated for the evaluation of the interreader reliability and intrareader reproducibility of feature extraction. A total of 200 cases of PET images randomly selected from the whole data were drawn by the ROIs by Reader 1 and Reader 2. After 2 weeks, Reader 1 repeated the segmentations. An ICC of greater than 0.75 denoted a favorable agreement of feature extraction. The ROI segmentation for accessing was performed by Reader 1. Second, maximum relevance and minimum redundancy (mRMR) and the 10-fold cross-validated least absolute shrinkage and selection operator (LASSO) method were employed for the radiomic feature selection from the training set (24). The utility of the LASSO regression model begins with the identification of optimal coefficient lambda (λ) among a multitude of radiomic features. By adjusting λ, LASSO could differentiate signatures that do not associate with PM by shrinking their coefficients to zero. Subsequently, the rest of the signatures with a nonzero coefficient are selected for the establishment of a radiomics score. A radiomics signature was generated *via* a linear combination of selected features weighted by their respective coefficients. All features extracted from the LIFEX software are shown in **Supplementary Material**.

### Construction of the Model and Clinical Utility

Clinical characteristics (gender, age, alcohol, smoking), symptoms (abdominal pain, fever, vomit), complication (weight loss), laboratory data (CEA, CA199, CA125), and PET parameters (SUVmax, SUVmean, MTV, TLG) were compared by Mann–Whitney U-test. Clinical variables and PET parameters analyzed by using univariate analysis of the training set with statistical significance (P<0.05) were selected into a multivariable logistic regression analysis using backward stepwise selection. Based on the selected covariates, a radiomics nomogram was then constructed. The nomogram was used to provide a visual tool for clinical use. In order to assess the discrimination performance of established models, we determined the area under the curve (AUC) of the receiver operating characteristic (ROC) curve. The net benefit of the predictive models was performed by the decision curve analysis (DCA) under different threshold probabilities to evaluate the clinical effectiveness of the nomogram (25).

### Statistical Analysis

All data analyses were performed using IBM SPSS (version 22.0) and R software (version 3.6.3). Numerical variables were compared by t test or Mann–Whitney U test, and categorical variables were analyzed by using χ^2^ test or Fisher’s exact test. Univariate and multivariate Cox regression analyses were performed to determine the predictors of PM (+) and PM (-). Multivariate analysis was applied to all variables with P value < 0.05 in univariate analysis. And P value < 0.05 was considered to indicate statistical significance.

## Results

### Demographic and Clinical Characteristics

A total of 355 eligible patients were selected for our study. Patients were randomly divided into a training set and a validation set according to the 7:3 ratio. A total of 250 patients were assigned to the training set and 105 patients to the validation set. The detailed characteristics of the patients are summarized in **Table 1**.

**Table 1:** Demographic and clinical characteristics of the study population.

	Training cohort			Validation cohort		
	GC-PM (+)	GC-PM (−)	*P* value	GC-PM (+)	GC-PM (−)	*P* value
**Characteristic**	(n=77)	(n=173)		(n=32)	(n=73)	
Age, mean (SD)	64.3 (12.3)	67.2 (11.3)	0.32	65.5 (14.6)	66.4 (11.8)	0.74
Gender (F/M)	25/52	44/129	0.07	9/23	18/55	0.89
Smoking	10 (12.9%)	45 (26.0%)	0.03	9 (25.00%)	25 (32.89%)	0.40
Alcohol	9 (11.7%)	38 (21.9%)	0.03	7 (19.44%)	19 (25.00%)	0.52
Gastric ulcers	8 (10.4%)	25 (14.5%)	0.20	3 (8.33%)	11 (14.47%)	0.36
Symptoms						
Abdominal pain	45 (58.4%)	97 (56.1%)	0.96	26 (81.3%)	41 (56.2%)	0.07
Fever	3 (3.9%)	6 (3.5%)	0.64	2 (6.3%)	2 (2.7%)	0.44
Vomiting	6 (7.8%)	30 (17.3%)	0.07	10 (31.3%)	9 (12.3%)	0.04
Weight loss	19 (24.7%)	40 (23.1%)	0.90	14 (43.8%)	15 (20.5%)	0.03
Laboratory						
CA 19-9 (U/ml), mean (SD)	468.8 (1,574.5)	510.2 (2,427.7)	0.89	1,256.3 (3,740.4)	308.6 (1,226.1)	0.06
CEA (μg/L), mean (SD)	41.7 (197.6)	130.1 (997.2)	0.44	442.6 (1,903.7)	87.6 (547.9)	0.14
CA 125 (U/ml), mean (SD)	221.2 (492.4)	53.9 (113.3)	<0.01	437.7 (998.5)	95.1 (240.5)	<0.01
SUV_Mean_, mean (SD)	4.1 (1.9)	4.6 (2.2)	0.37	4.4 (2)	4.5 (2.1)	0.37
SUV_Max_, mean (SD)	7.4 (4.7)	10.1 (14.3)	0.05	8.4 (5.3)	9.2 (12.3)	0.03
MTV, mean (SD)	4.4 (5.9)	3.7 (4.1)	0.34	4.6 (11.8)	3.9 (4.7)	0.27
TLG, mean (SD)	21.9 (45.8)	21.5 (40.8)	0.95	26.4 (84.9)	21.6 (42.3)	0.31
Radiomic Score, median [iqr]	-0.9 [-1.5, -0.1]	1.7 [0.2, 3.4]	<0.01	-0.5 [-1.4, 0.5]	2.4 [0.9, 4.0]	<0.01

### Feature Selection and Radiomics Signature Building

**Figure 3:**
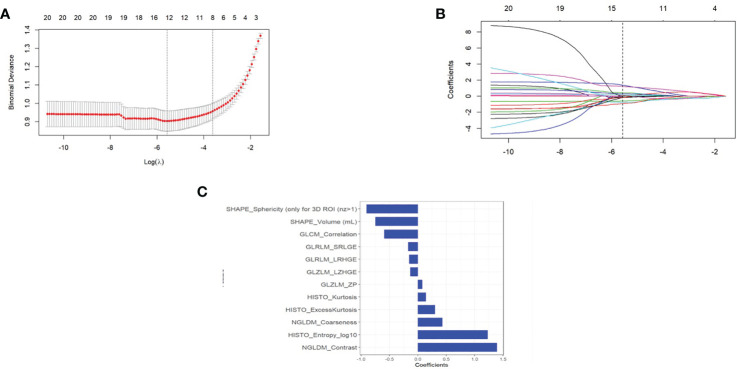
**(A)** The error rate curve. **(B)** LASSO coefficient λ graph. We chose the coefficient λ with the lowest error rate. **(C)** The remaining features of the positron emission tomography (PET) images after feature selection.

A total of 69 texture features were extracted from PET images for each patient. After removing the features with an ICC ≤0.75 through performing mRMR and LASSO logistic regression analysis, the optimized subset of features were left to construct the final model (**Figure 3**).

Radiomics signature = 0.589*GLCM_Correlation+1.393*NGLDM_Contrast-0.747*SHAPE_Volume(mL)+1.23*HISTO_Entropy_log10-0.133*GLZLM_LZHGE+0.078*GLZLM_ZP+0.432*NGLDM_Coarseness+0.141*HISTO_Kurtosis-0.17*GLRLM_SRLGE-0.151*GLRLM_LRHGE +0.303*HISTO_ExcessKurtosis-0.905*SHAPE_Sphericity (only for 3D ROI (nz>1) + 1.068

**Figure 4:**
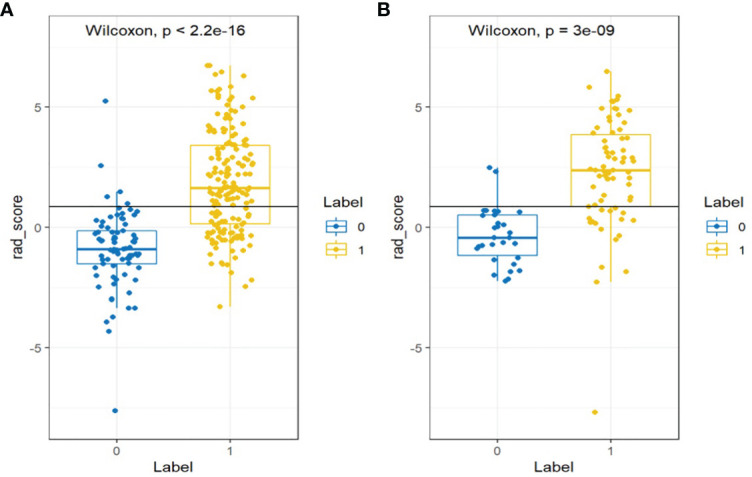
Wilcoxon analysis of Rad-score for detecting the PM of GC in the **(A)** training cohort and **(B)** validation cohort (p < 0.05).

The differences in the Rad-score value between the negative and positive PM in the training and validation cohorts were statistically significant (**Figure 4**).

### Diagnostic Validation of Radiomics Signature Building

**Figure 5:**
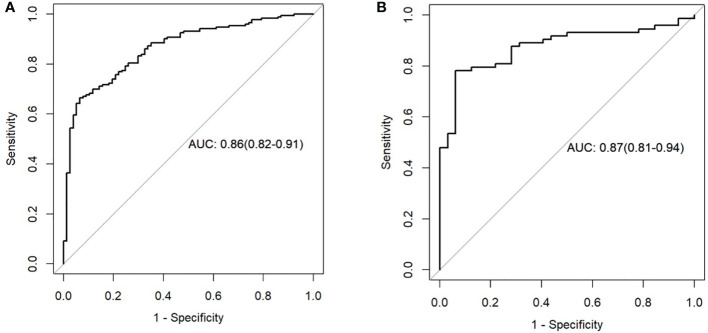
Receiver operating characteristic (ROC) curves of the radiomics model in the training set **(A)** and testing set **(B)**.

**Figure 6:**
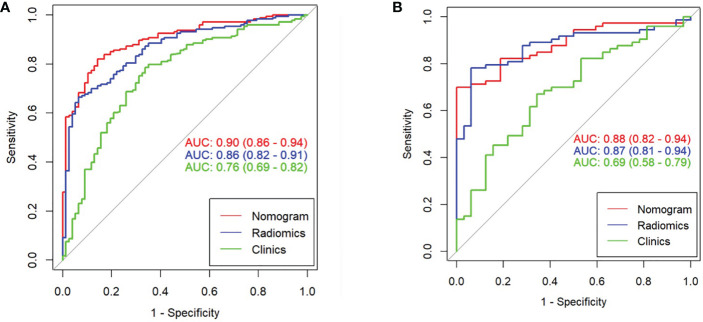
Comparison of ROC among the nomogram, radiomics model, and clinical model for the prediction of GC-PM in the **(A)** training and **(B)** validation cohorts.

The clinical prediction model was based on CA125 and SUVmax of 0.76 [95% confidence interval (CI): 0.69,0.82] and 0.69 [95% confidence interval (CI): 0.58, 0.79], respectively. The radiomic model showed significantly better discriminative ability (P < 0.05) than the clinical model for predicting the PM of GC with the AUC of 0.86 [95% confidence interval (CI): 0.82,0.91] and 0.87 [95% confidence interval (CI): 0.81, 0.94] in the training and validation cohorts, respectively (**Figures 5A, B**). Furthermore, a comprehensive model combined clinical factors (SUVmax, CA125) with the radiomic signature together had AUCs of 0.90 [95%CI, 0.86–0.94] and 0.88 [95% confidence interval (CI): 0.82, 0.94] in the training and validation cohorts, respectively (**Figures 6A, B**). However, the comparison of AUCs between the radiomic model and the comprehensive model showed no significant difference (P>0.05), which indicated that the radiomic signature plays a significant role in predicting the PM of GC.

### Clinical Use

**Figure 7:**
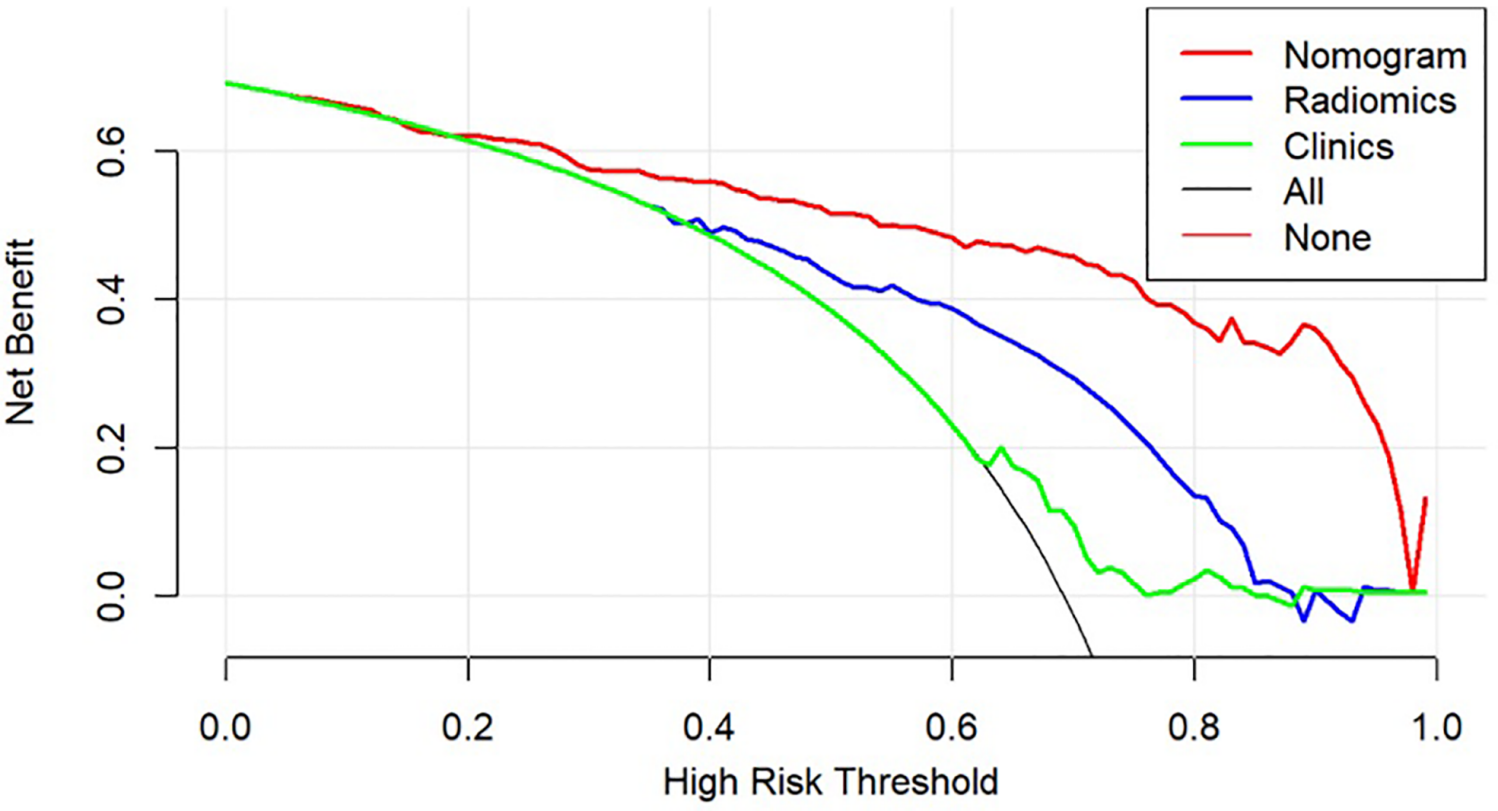
Decision curve analysis for models. The y-axis measures the net benefit, which is calculated by summing the benefits (true-positive findings) and subtracting the harms (false-positive findings), weighting the latter by a factor related to the relative harm of undetected GC-PM(+) compared with the harm of being mistaken for GC-PM(-).

The DCA was used to compare the benefit of the radiomic nomogram, the clinical prediction model, and the comprehensive model, which are presented in **Figure 7** (26).

## Discussion

The purpose of this study was to evaluate the value of a computer-assisted method derived from a great quantity of clinical and PET data to preoperatively predict the PM in patients of GC. We found that the comprehensive model integrating Rad-score, SUVmax, and CA125 had a promising predictive value for the PM of GC patients. Moreover, the model can provide a tool to assist clinicians to predict PM noninvasively.

The peritoneum is the most probable position of distant metastasis in GC (27), and PM is proven to independently affect prognosis in patients with GC (28). Once peritoneum metastasis is found, preoperative and postoperative chemotherapy and chemoradiation therapy are effective in prolonging relapse-free survival and overall survival (29–31). To the point of clinical view, it is crucial to preoperatively predict the PM of GC patients in order to select the proper treatment strategy. There are many ways to detect the PM of GC patients. Detecting the PM status through performing laparoscopy was the golden criterion (32). However, it is not suitable for each patient because of its invasive diagnostic procedure. Besides, it limits the possibilities of information from the spatiotemporal heterogeneity of tumors (20, 33). For another way, the accuracy for discriminating the PM status by using CT was very limited in patients with GC (34). PET/CT was a good tool that had a great value on distant organ metastasis (35), and Findlay et al. (36) also mentioned that ^18^F-FDG-PET/CT could provide useful information in identifying unsuspected metastasis. Meanwhile, Smyth et al. (37) carried out a study of 113 locally advanced gastric cancer patients and pointed out a 10% reduction in the number of ineffective procedures after performing ^18^FDG-PET/CT.

The information obtained from the noninvasive conventional images is finite, while a great deal of valuable data remains concealed in the images (14, 38). Recently, radiomics has been proven to be an indispensable diagnostic tool to identify histological and biological characteristics of tumors beyond visual assessment on conventional CT, PET/CT, and MRI images. Tang et al. (39) pointed out that the radiomic nomogram can greatly and effectively estimate early recurrence risks of resectable pancreatic cancer patients preoperatively. Moreover, Wang et al. (40) established a nomogram with promising results, which can assess an individual risk and provide guide treatment decisions for patients.

Therefore, the nomogram is a statistical model that provides a useful and meaningful method for doctors. However, no study has used the radiomic approach, which was based on the PET, to predict the PM of GC. Our PM-related radiomics signature performed excellent predictive ability and was an independent predictor of GC. During the construction of the radiomics signature, we got the more stable radiomics features such as Shape_Sphericity [only for 3D ROI(nz>1)] and NGLDM_Contrast. These two texture features have also been reported in some other tumors. The NGLDM reflects the difference of the gray level between one voxel and its 26 neighbors in three dimensions. Xu H et al. (41) pointed out that the NGLDM presented more significant differences between hepatocellular carcinoma and hepatic lymphoma. Yu T et al. (42) indicated that the AUC value improved when the shape_sphericity feature was combined with SUVmax. These results are entirely consistent with our research.

In our study, 12-feature radiomics signature and 2 factors (CA125 and SUVmax) are integrated in our radiomics nomogram. Our results gained outbreaking progress compared with a previous study. According to the nomogram (AUC 0.90) in the training set and (AUC 0.88) in the validation set, the accuracy of the prediction was obviously higher than the investigation that was based on CT with an AUC of 0.75 (43). In contrast to CT, PET has better performance, which means metabolic parameters play an important role in the model. The most commonly used metabolic parameter of PET/CT is SUVmax, which indicates the additional value of predicting metastasis and prognosis (44, 45). In addition, CA125 is one of the clinically promising diagnostic markers in evaluating the efficacy of chemotherapy and predicting the prognosis of patients with peritoneal dissemination (46). Therefore, our results could comprehensively reflect the PM of GC, and patients would benefit from treatment guidance.

On the other hand, this study still has several limitations. Firstly, this is a retrospective study; the potential selection bias cannot be excluded. Secondly, the pathological stage of the tumor was not included in this study. Diagnosing GC in stages T3–T4, patients are more likely to have a higher risk of PM (47). Thirdly, the deviation of the results may be caused by irregular lesions. Fourthly, although the features of CT are valuable for predicting peritoneal metastasis, our radiomics model was only developed by preoperative positron emission tomography (PET). A comprehensive model will be constructed in our future study, which will combine CT with PET radiomics signatures. Lastly, the models were established based on a small sample in a single institution and also did not refer to prognosis. Therefore, prospective studies should be explored with further validation by multiple centers’ clinical trials; prognosis is also the next step of our research work.

## Conclusion

In conclusion, we established a radiomics model based on ^18^F-FDG PET integrating clinical and metabolic factors to predict the PM of GC. We found that the comprehensive model with satisfied diagnostic performance can be recommended as a potential method for predicting the PM status in GC patients preoperatively.

## Data Availability Statement

The datasets presented in this study can be found in online repositories. The names of the repository/repositories and accession number(s) can be found in the article/**Supplementary Material**.

## Ethics Statement

The studies involving human participants were reviewed and approved by the First Affiliated Hospital of Wenzhou Medical University. Written informed consent for participation was not required for this study in accordance with the national legislation and the institutional requirements. Written informed consent was not obtained from the individual(s) for the publication of any potentially identifiable images or data included in this article.

## Author Contributions

KT conceived the idea of the study. BX and JJ collected the data. LC, SW, and XZ performed image analysis. BX wrote the manuscript and performed the statistical analysis. BX, KT, and XWZ edited and reviewed the manuscript. All authors contributed to the article and approved the submitted version.

## Conflict of Interest

The authors declare that the research was conducted in the absence of any commercial or financial relationships that could be construed as a potential conflict of interest.

## Publisher’s Note

All claims expressed in this article are solely those of the authors and do not necessarily represent those of their affiliated organizations, or those of the publisher, the editors and the reviewers. Any product that may be evaluated in this article, or claim that may be made by its manufacturer, is not guaranteed or endorsed by the publisher.

## Abbreviations

GC, gastric cancer; PM, peritoneal metastasis; PM (+), peritoneal metastatic positive; PM (-), peritoneal metastatic negative; CT, computed tomography; PET, positron emission tomography; mRMR, maximum relevance and minimum redundancy; LASSO, least absolute shrinkage and selection operator; DCA, decision curve analysis; ROI, region of interest; ICC, intraclass correlation coefficient; AUC, area under the curve; H-L, Hosmer–Lemeshow; WMU, Wenzhou Medical University; CEA, carcinoembryonic antigen; CA125, carbohydrate antigen 125; CA199, carbohydrate antigen 199; SUVmax, maximum standardized uptake value; MTV, metabolic tumor volume; TLG, total lesion glycolysis; NGLDM, neighborhood gray-level different matrix; GLCM, gray-level co-occurrence matrix; GLZLM, gray-level zone length matrix; GLRLM, gray-level run length matrix.
